# Guidelines for Negotiating Scientific Collaboration

**DOI:** 10.1371/journal.pbio.0030217

**Published:** 2005-06-14

**Authors:** Neil R Smalheiser, Guy A Perkins, Steve Jones

## Abstract

Whether it's sharing reagents with a laboratory on the other side of the world or working with the postdoc at the neighboring bench, some simple rules of collaboration might help.

The scientific enterprise reflects the willingness of scientists to collaborate implicitly with people they don't know personally: investigators routinely obtain antibodies, enzymes, sterile supplies, and other items from commercial suppliers without hardly blinking, and often without knowing anything about the companies other than what appears on the sales brochures. This system works because of basic warranties and expectations, as well as competition among companies to maintain high quality standards and easy, quick availability of specialized reagents. It is no exaggeration to say that vendors, as de facto scientific collaborators, are a major driving force in scientific productivity.

In contrast, many scientists are far more reluctant to enter into explicit collaborations with other academic scientists, even those who are well respected and well established, unless they have a strong prior personal relationship with them. Why?

There are many concerns that a scientist might have that could outweigh the potential advantages of collaboration with another academic. Unlike a commercial vendor, whose participation is passive and circumscribed, an academic collaborator is likely to argue passionately about the design of the experiments, may not agree with the underlying hypothesis, might engage in experiments that lack certain perceived controls or quality standards, or may simply lose momentum (e.g., if the lab loses grant support, or if a student in the lab leaves who was doing the experiments). Even after the basic experiments are finished, an academic collaborator might delay or even prevent publication by insisting on the need for further experiments, proper credit, a certain author name order, having intellectual property rights, submitting to a certain journal, and so on. Doing scientific research is a highly personal and subjective activity, so that it is difficult to put two investigators together at random and expect them to see eye-to-eye. Also, each member of the collaboration is privy to valuable unpublished data, facts, ideas, and hypotheses that they are loathe to disclose to the other without good reason. Making a commitment to collaborate fully with another laboratory is far from a trivial decision.

Thus, collaborations tend to fall into one of two categories at opposite ends of the spectrum: on the one hand, the passive or one-sided vendor model, where a person supplies a reagent with minimal warranties and expectations; and on the other hand, the active collaborator model, where two or more investigators are fully engaged in a common pursuit with full sharing of ideas and credit (think Watson and Crick). This leaves an enormous set of potential opportunities in the middle, consisting of limited collaborations that could be mutually fruitful, but that often cannot get started or be sustained because of uncertainty and lack of trust. We suggest that it is possible to encourage more of these limited collaborations to proceed by providing a list of the key points to be considered at the onset of a collaboration.

What we have in mind is not along the lines of a legal contract; rather, we envision a set of voluntary “boilerplate” guidelines that two academic biomedical investigators, who are potential collaborators, can refer to as a reference point for negotiation. This would not threaten the current informal way that scientists tend to enter collaborations. Nor would the use of these guidelines incur any obligations on the universities or other institutions that employ the scientists involved.

Although minimal guidelines for sharing of reagents and data have been widely discussed (e.g., [1–6]), to our knowledge, no one has explicitly enumerated the points of collaboration that should be negotiated between two academic biomedical investigators. Here, we propose a set of possible guidelines (Box 1). We confine the terms to collaborations between a supplier (of reagents, expertise, specialized equipment, data, methods, or computer code) and a receiver, who is often the initiator of the collaboration. However, the guidelines could apply to collaborations generally, regardless of the nature or direction of sharing involved, and regardless of whether the collaboration is formalized (e.g., as in a contract for services) or is pursued as an informal verbal agreement. As negotiations of intellectual property issues generally involve additional parties such as host institutions and funding agencies and are not solely under the control of the two collaborators, a detailed consideration of such issues falls beyond the scope of the present guidelines.

**Box 1. Suggested Guidelines for Negotiating Scientific Collaboration**

The guidelines here are discussed in terms of a collaboration involving a supplier (e.g., of reagents, expertise, specialized equipment, data, methods, or computer code) (Party 1) and a receiver (Party 2). The options in each section are listed in order of increasing involvement by Party 1 in the collaboration.

1. **Sharing of reagents and data.**
  - **Minimal:** Reagents or data will be provided that are essentially as described in published description, and Party 1 warrants that these will be prepared, stored, and shipped in a manner that will preserve their value. Party 2 will pay the costs of shipping. If the reagent is not readily available in the supplier's laboratory and there are no plans to prepare more, Party 2 may be asked to share reasonable costs of preparing the reagent for shipping.
  - **Option 1:** Party 1 will give all available information, based on unpublished results, to help the receiver save time and use the reagents in an optimal fashion. (For example, when supplying an antibody for immunohistochemistry, the supplier will suggest a proven range of dilutions, buffers, and fixatives.)
  - **Option 2:** Both parties will describe the nature of unpublished information related to ongoing experiments in their respective laboratories, in sufficient detail that the parties can decide whether it is mutually to their benefit to share unpublished information, in whole or in part.
2. **Design of experiments.**
  - **Minimal:** Party 1 will not be privy to details of the intended use of the reagents or data, beyond those necessary to prepare the reagents adequately.
  - **Option 1:** Party 2 will describe the design of their experiments and invite comments from Party 1. Any suggestions that are implemented will be considered part of an active collaboration and credited appropriately.
  - **Option 2:** Both parties will contribute actively to the design of experiments, including control experiments.
3. **Division of labor.**
  - **Minimal:** Party 1 provides reagents/expertise/equipment/data but does not participate in the experiments of Party 2.
  - **Option 1:** Party 1 provides training and specialized expertise to personnel of Party 2, thus facilitating the work, but does not carry out the research directly.
  - **Option 2:** Both parties participate in experiments, but each does the subset of experiments they are most experienced, knowledgeable, or comfortable with and the other group(s) do the same with nonoverlapping subsets of experiments.
  - **Option 3:** Party 1 provides assistance with personnel and training to help with Party 2's experiments, forming an interlaboratory team that has ongoing communication during experiments and that shares the costs of doing the research.
4. **Publication of results stemming from the collaboration.**
  - **Minimal:** Party 2 will acknowledge the source of the reagent or data in their publications, and will cite the providers' relevant publications that described the reagent and its use.
  - **Option 1:** Party 1 will be given a copy of the manuscript prior to publication and given the option to be included in the list of authors, unless Party 1 disagrees materially with the paper, or fails to answer in a reasonable time frame.
  - **Option 2:** Both parties participate in writing the paper, and the resulting publication spells out the contributions of each.
5. **Co-authorship order.**
  - **Minimal:** Party 2 will write the paper and choose the order of authorship that Party 2 feels is fair and appropriate.
  - **Option 1:** At the time that a decision is made to offer co-authorship to Party 1, Party 2 will discuss the planned order of authors and the rationale with Party 1.
  - **Option 2:** As the experiments are being designed and planned, both parties will be apprised of the relative roles of individuals in both laboratories, and a tentative co-author list will be discussed. Any changes will be discussed in advance of writing the paper.
6. **Access to unpublished data arising from the collaboration.**
  - **Minimal:** Party 1 has no right to access to any data obtained by Party 2.
  - **Option 1:** Party 2 will give basic feedback on how the material supplied by Party 1 was used.
  - **Option 2:** Party 2 shares the specific results obtained with Party 1, with the understanding that such information is confidential and cannot be used by Party 1 without the written consent of Party 2.
  - **Option 3:** Party 1 has access to data originally intended for publication under joint authorship, whether or not the data are actually published. The unpublished data can be used by Party 1 in their grant submissions and for their own knowledge.
  - **Option 4:** Both parties will discuss the types of experiments that are being conducted in the general area of the collaboration, and will discuss which activities (beyond the joint experiments) should be shared knowledge.
7. **Intellectual property issues.** Negotiations of intellectual property issues fall beyond the scope of the present guidelines. However, potential collaborators should at least acknowledge the types of intellectual property issues that may arise in the course of the collaboration.
  - **Minimal:** Party 2 does not share in any intellectual property related to the existing reagent or data.
  - **Optional:** If the joint experiments or data analyses result in new information or uses having commercial value, both parties will negotiate shared intellectual property.

These guidelines will be posted on the Science Commons Web site (http://science.creativecommons.org/) to provide a public forum encouraging scientific collaborators and others to provide feedback and suggestions, and allowing the guidelines to be modified and extended over time.
